# Multifunctional resin-matrix ceramic: synergistic mechanical–biological optimization and novel strategies for translational research

**DOI:** 10.1039/d5ra02325d

**Published:** 2025-07-07

**Authors:** Jingsong Mao, Mingkai Wang, Jianhua Liang, Chengde Jin, Hanbo Zhang, Qiang Wang, Zhuoqun Yan, Yuzhong Gao, Tao Yan

**Affiliations:** a Liaoning Provincial Key Laboratory of Oral Diseases, School and Hospital of Stomatology, China Medical University Shenyang 110001 China; b Department of Stomatology, Xiangyang No. 1 People's Hospital, Hubei University of Medicine Xiangyang Hubei 441000 China; c Liaoning Upcera Co., Ltd Benxi 117004 China; d The First Affiliated Hospital of Jinzhou Medical University Jinzhou Liaoning 121001 China; e Xiangyang Hospital Affiliated to Hubei University of Chinese Medicine Xiangyang Hubei 441000 China yant202412@163.com

## Abstract

The advancement of dental materials has established resin–zirconia RMC (resin-matrix ceramic) as a pivotal innovation in restorative dentistry, combining zirconia's mechanical strength with resin's elasticity to overcome the limitations of traditional systems. Conventional materials often compromise tooth integrity due to elastic modulus mismatch and cytotoxic monomer release, whereas resin–zirconia RMC (resin-matrix ceramic) achieve stress distribution aligned with natural dentition while enhancing biocompatibility. This review explores their design strategies, including nano-zirconia reinforcement, polymer–ceramic network optimization, and surface functionalization, which collectively improve wear resistance, aging stability, and antibacterial efficacy. Clinically, these composites demonstrate exceptional performance, with long-term success and minimal wear under cyclic loading. Mechanistically, they regulate cellular interactions critical to soft tissue healing and bone integration, suppressing inflammatory pathways while promoting osteoblast activity and collagen alignment. Despite these advancements, challenges such as the long-term biocompatibility of wear particles and processing complexity require further investigation. By integrating material science, cell biology, and clinical insights, this work underscores the potential of resin–zirconia RMC (resin-matrix ceramic) to redefine restorative dentistry through harmonized mechanical and biological functionality.

## Abstract 2

The advancement of dental materials has led to resin–zirconia RMC (resin-matrix ceramic), a major step forward, combining zirconia's strength with resin's flexibility to overcome traditional limits.

## Graphical abstract

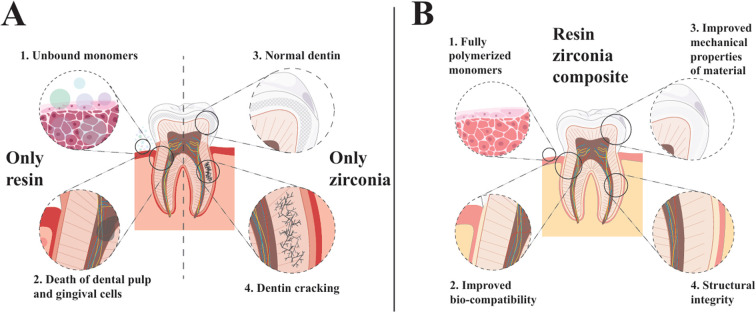

## 1 Introduction

Resin materials, used for over six decades, have seen remarkable progress alongside the continuous evolution of the dental industry and expanding research. Due to their versatility as direct restorative materials and ease of therapeutic manipulation,^1^ they are commonly used in clinical settings to address complex prosthodontic challenges. However, the safety of resin-based materials often fails to meet the clinical requirements. Dental resins release free monomers in the human oral environment when they undergo polymerization.^2^ Over time, resin degrades due to the esterase activity in the oral cavity, leading to the breakdown of commercial resin materials.^3^ Breakdown products are liberated in their monomeric state when exposed to water or other solvents. Unbound monomers exhibit cytotoxic effects on pulp and gingival cells and may induce allergic reactions in the body.^4,5^ When people brush their teeth daily, the interaction of fluoride ions from toothpaste with resin produces reactive oxygen species (ROS), resulting in cell death due to tissue damage.^6^

Zirconia materials are gaining distinction due to their superior biocompatibility and safety compared to other options.^7^ Additionally, zirconia meets the standards for implant restoration in terms of physical properties, aesthetics, and corrosion resistance.^8^ These noticeable properties make monolithic zirconia a preferred choice for posterior tooth crowns.^8^ Despite the increase in studies, significant problems and potential challenges in clinical application remain. The main problem is that zirconia materials have a higher elastic modulus than teeth and bone tissue.^9^ Owing to their large elastic modulus, the material causes stress concentration in the dentin, forming a region of high stress and causing dentin cracking.^10^ This problem creates substantial resistance and difficulties in clinical applications. Therefore, enhancing the mechanical properties of zirconia materials to match those of human hard tissue has become a key goal in dental material research.

**Fig. 1:**
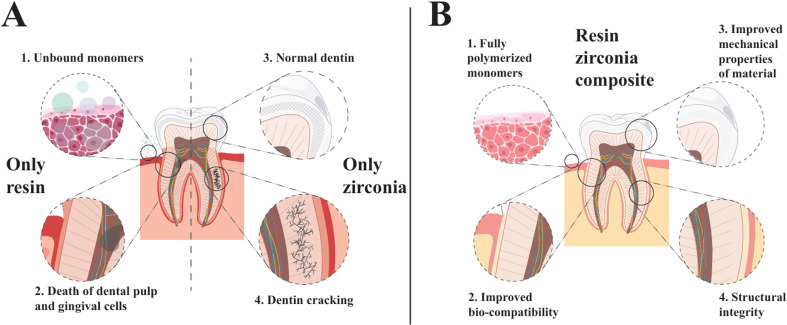
Comparison of zirconia materials in oral applications: (a) drawbacks of employing resin or zirconia alone in oral repair; (b) benefits of using resin–zirconia RMC (resin-matrix ceramic).

Therefore, the development of composite materials with multiple advantages is a hot topic. Among the materials currently used in oral restoration, resin has the most potential to compound with zirconia.^7^ One reason is that the hardness and elastic modulus of resin match well with those of bone tissue^11^ compensating for zirconia's deficiency. Simultaneously, by changing the proportion of zirconia to resin, the biocompatibility and mechanical properties of the materials can be increased.^12^ However, resin–zirconia RMC (resin-matrix ceramic) still require extensive performance evaluation and clinical applications for better development,^13^ as shown in Fig. 1.

This review comprehensively discusses the current research progress in the performance and clinical applications of resin–zirconia RMC (resin-matrix ceramic). The wear resistance, biocompatibility, and other clinical characteristics were evaluated. This review mainly expounds on the advantages of combining resin and zirconia, the treatment for prolonging the service life of the composite and evaluating the characteristics of the composite based on existing data. The factors affecting the properties of these materials were also analyzed, offering new prospects for resin–zirconia RMC (resin-matrix ceramic) research.

## 2 Performance and evaluation of resin–zirconia RMC (resin-matrix ceramic) materials

### 2.1 Mechanical performance: elastic modulus and fracture toughness

In addition to elastic modulus and fracture toughness, three core properties are routinely reported for dental restorative materials: flexural strength (resistance to bending forces), compressive strength (ability to resist masticatory compression) and Vickers hardness (surface hardness linked to wear). Clinically, a flexural strength >100 MPa, compressive strength >300 MPa and Vickers hardness within 2–4 GPa are considered acceptable for posterior restorations. These metrics contextualize the resin-matrix ceramic data summarized below.^14,15^

When applied to the oral cavity, dental materials must withstand substantial and complex bite forces. Additionally, the temperature and hardness of food also affect their performance. Dental materials must exhibit outstanding mechanical, particularly dynamic, properties. Researchers can categorize mechanical properties into various categories, such as elastic modulus and fracture toughness. The elastic modulus measures a material's ability to absorb stress and transfer loads, influencing stress distribution in dental roots.^14^ Proper stress distribution and load transfer are key to the long-term success of restorations.^15^ The elastic modulus of the human enamel is between 48 and 105.5 GPa. When the elastic modulus of a restorative material exceeds this range, its capacity to absorb stress declines significantly, causing stress concentration. As a result, low-strength dental tissues can fracture under excessive impulse force,^16^ including dentin cracking.^17^ When the elastic modulus of the restorative material is much lower than this value, it cannot assist a substitute for the periodontal ligament to absorb, transduce, and disperse the occlusal loads,^18^ causing the treatment failure. However, when the elastic modulus of the restorative material is close to this value, it breaks before the tooth tissue with an excessive load, which can reduce the probability of odontoclasia.^19,20^ Thus, dental materials are required to approximate the elastic modulus of the human dental hard tissue. The elastic modulus of the resin–zirconia RMC (resin-matrix ceramic) is slightly lower than this range, while that of one material, the polymer-infiltrated ceramic network (PICN), is closer to it. Its elastic modulus is between 41.3 and 99.3 GPa.^16^ Hence, compared with other materials, the resin–zirconia RMC (resin-matrix ceramic) material has more significant advantages. It can provide better protection for the periodontal tissue and increase the probability of long-term success.

Fracture toughness, another parameter closely related to the quality of materials, is also known as the critical stress intensity factor (*K*). It depends on applied stress, geometry, and crack size. With increased applied stress, *K* increases to a critical point (*K*_c_) at which fast crack propagation occurs, and the material undergoes catastrophic failure.^16^ Hence, under long-term chewing and non-axial loads, the interior of the material with poor fracture toughness appears as a small crack. The growth of these cracks disrupts the interface between the base material and the filler, ultimately leading to material rupture and repair failure.^17^ Despite the challenges, the advantage of one-step implant surgery makes one-piece implants a viable option for many patients. According to a systematic review, one-piece zirconia implants have a 94.4% survival rate and 91.6% success rate over 3 years, with acceptable marginal bone loss and favorable biological outcomes.^18^ In clinical prospective studies, the 3-year survival rate of one-stage zirconia implants was 98.5% and showed a trend of low marginal bone loss.^19^ The use of resin ceramic abutments can continue to support the survival of such implants and have good clinical applications. However, the resin–zirconia RMC (resin-matrix ceramic) can increase its fracture toughness by the bridge fiber of the resin and cause the crack to branch and deflect, reducing the likelihood of rupture and achieving clinical indicators.^20^

Due to varying proportions of resin and zirconia, the mechanical properties of these composites differ slightly. The mechanical properties of the resin–zirconia RMC (resin-matrix ceramic) were improved compared with those of the resin composite without zirconia.^21^ Polymethyl methacrylate (PMMA) is a standard resin substrate. This often results in fractures of the denture base.^22^ However, the incorporation of nanometer zirconia (nano-zirconium) into PMMA can improve its function, significantly increasing its bending strength and fracture resistance. The finding shows that at 3% nano-ZrO_2_, bending strength peaked (94.42 *vs.* 87.54 MPa control), attributed to crack deflection *via* SEM/FTIR, while excessive concentrations (5%) induced stress concentration (*p* < 0.05, ASTM D790 tests), aligning with optimal mechanical neutralization for dental PMMA.^23^

### 2.2 Adhesion performance

Zirconium has been introduced for dental use as a core material for conventional or resin-bonded fixed partial dentures and complete coverage crowns because of its superior mechanical properties compared to those of traditional ceramic materials. Currently, the most accepted method for enhancing adhesion in zirconia composite materials involves using resin adhesives and surface treatments.^24^ The 10-methacryloyloxydecyl hydrogen phosphate (MDP) phosphate monomer with a bonding agent can bond with the zirconia surface *via* a chemically stable covalent bond (–P–O–Zr–) to enhance the adhesion performance, especially after sandblasting.^25,26^ This chemically stable bond is crucial for resin–zirconia RMC (resin-matrix ceramic) to resist damage caused by thermal expansion and contraction during thermal cycling.

Sandblasting plays a key role in enhancing adhesion between zirconia and resin composites.^26^ However, the hardness of zirconia is so high that coarsening after sandblasting cannot achieve the ideal effect. It has been proven that the hardness of resin–zirconia RMC (resin-matrix ceramic) will decrease significantly by adding the resin to the zirconia.^27,28^ This makes the material more receptive to sandblasting, improving surface wettability, increasing surface energy, and creating a rougher surface on the resin–zirconia RMC (resin-matrix ceramic).^26^ The adhesive penetrates the ceramic surface more effectively, further strengthening the micromechanical mosaic.^29^ At the same time, it enhances the hydrophilic properties of the resin–zirconia RMC (resin-matrix ceramic). The resin phase within the composite further strengthens these hydrophilic properties, contributing to improved bonding strength.^30^ These advancements in adhesion and material optimization are reflected in clinical outcomes. A 3-year study of PICN single crowns reported a 93.9% survival rate and 92.7% success rate, with no debonding observed, underscoring the long-term reliability of resin-matrix ceramic (RMC) systems in restorative dentistry.^31^

In current research on adhesive,^32,33^ investigations predominantly focus on the effects of surface treatment modalities on bond strength. Established approaches include hydrofluoric acid (HF) etching combined with salinization, sandblasting (airborne-particle abrasion with Al_2_O_3_ particles) followed by salinization, and sole sandblasting. It has been well-established that HF etching coupled with silanization represents a validated strategy to enhance the micro shear bond strength (μSBS) of resin cement to polymer-infiltrated ceramic network (PICN) materials, underscoring the pivotal role of silane coupling agents in interfacial adhesion. Nevertheless, sandblasting with subsequent silanization or universal adhesive application provides a clinically viable alternative, particularly when HF application is clinically contraindicated. Future studies should prioritize long-term validation of treatment efficacy across diverse protocols and novel adhesive systems, alongside material-specific analyses to elucidate the differential responses of distinct ceramic substrates (*e.g.*, PICN *vs.* resin nanoceramics). Such efforts will refine evidence-based clinical guidelines for optimizing durable adhesive interfaces in restorative dentistry.

#### 2.2.1 Interfacial chemistry between resin and zirconia

The long-term success of resin-matrix ceramics (RMCs) is governed by the stability of the silica–zirconia–resin interface that forms during chairside conditioning. After airborne-particle abrasion a tetragonal-zirconia surface is covered with Zr–OH groups. Three sequential steps occur: (1) surface hydroxylation, (2) silanisation with γ-methacryloxypropyl-trimethoxysilane (MPTS), and (3) co-polymer grafting with Bis-GMA/UDMA. Silanols condense with Zr–OH to create Zr–O–Si bonds and leave a methacrylate tail that co-polymerizes with the resin. Density-functional calculations give a binding energy of −187 kJ mol^−1^ for these Zr–O–Si linkages, a value higher than that of equivalent Ti–O–Si bonds. If a 10-MDP primer is applied, phosphate groups chelate zirconia through mono-dentate (Zr–O–P) and bi-dentate (Zr2–O2–P) complexes; solid-state 31P NMR shows the latter dominates after 60 s of light activation and raises micro-tensile bond strength by roughly 35 percent. Finally, nano filled adhesive diffuses 1–2 μm into the porous ceramic network, producing an interpenetrating gradient layer (modulus 3–12 GPa) that reduces cyclic interfacial stress.^24,27^

#### 2.2.2 Polymerization mechanisms

RMC matrices cure by radical chain-growth polymerization of dimethacrylate blends (Bis-GMA, TEGDMA, UDMA) that infiltrate the pre-sintered ceramic. The manufacturer's dual-cure process combines: (a) camphorquinone–amine photoinitiation for surface conversion and (b) benzoyl peroxide–amine redox initiation at 110 °C under 0.6 MPa for bulk cure.^32,33^ Real-time FT-IR shows a biphasic conversion curve: a fast phase during the first 15 s (rate ≈ 0.18 s^−1^) and a diffusion-limited phase plateauing at 75–78 percent double-bond conversion. Adding 0.5 wt percent β-allyl sulfone (an addition–fragmentation chain-transfer comonomer) raises final conversion to about 85 percent without increasing polymerisation shrinkage. In zirconia-rich hybrids the ceramic surface acts as a radical sink; electron-paramagnetic-resonance studies reveal transient Zr–O˙ species that quench roughly 6 percent of radicals, so the initiator load is increased by 20 percent compared with PICN systems. A final glaze bake at 160 °C encourages residual initiator fragments to migrate to the surface, where routine polishing removes them and leaves monomer below 0.02 μg mm^2^.^28^

#### 2.2.3 Molecular mechanisms of material degradation

##### 2.2.3.1 Degradation of RMCs progresses through three coupled routes

###### 2.2.3.1.1 Hydrolytic ester cleavage

Water uptake (≈28 μg mm^−3^ after 30 days at 37 °C) plasticises the Bis-GMA network; accelerated ageing in 10 000 ppm NaOCl for 5 h lowers storage modulus by about 23 percent because ester bonds are cleaved, a process catalysed by leached tertiary amines.

###### 2.2.3.1.2 Zirconia low-temperature degradation (LTD)

Steam at 200 °C causes negligible t-to-m transformation (<1 percent) because the resin limits oxygen diffusion, but intra-oral electro-chemical cycling (pH 4–7.4, 106 cycles) produces a 0.6 percent t-phase loss and micro-crack densities of 0.4 mm^−1^. Silanes rich in Si–O–Zr cross-bridges slow this transformation three-fold.

###### 2.2.3.1.3 Enzymatic and oxidative attack

Salivary esterases cleave residual methacrylate side-chains; neutrophil-derived reactive oxygen species oxidise tertiary amines to nitroso-derivatives. LC-MS/MS detects Bis-GMA oxidation fragments (*m*/*z* = 515) after 21 days in activated macrophage medium. Nano-zirconia fillers scavenge hydroxyl radicals (rate constant ≈ 3.2 × 109 M^−1^ s^−1^), halving oxidative mass loss relative to filler-free controls.^27^

### 2.3 Abrasion resistance

Resin–zirconia RMC (resin-matrix ceramic) has become increasingly prevalent in dental restorations and prosthetic dentistry because of their superior physical and mechanical properties. These materials, praised for their abrasion resistances, are especially popular for restoring class I and II defects. While ongoing improvements have been observed, comprehensive longitudinal clinical investigations remain imperative to establish robust validation of their long-term clinical performance.

Abrasion, an inevitable result of long-term dental material use, occurs from the gradual loss of material due to the relative motion between contacting surfaces. Clinical studies conducted over extended periods have revealed that extensive posterior restorations, particularly in patients with parafunctional occlusal habits (such as clenching or bruxism), may exhibit suboptimal abrasion resistance.^34^ Clinical evaluations are crucial for assessing the wear performance of dental materials. However, such *in vivo* studies are often limited by high costs and variability in results owing to uncontrollable patient-related factors. *In vitro* studies, while useful, cannot fully replicate the multifaceted conditions of oral wear.^35^ We focused on research concerning the wear behavior of zirconia-based ceramics, summarized in Table 1.

**Table 1:** Resin–zirconia RMC (resin-matrix ceramic) testing overview

Materials	Evaluation	Observation	Findings	Ref.
PICN^a^	Reciprocating wear tests	PICN is less wear-resistant than tooth enamel but shares a comparable wear pattern	PICN shows greater wear than enamel but has fewer cracks in wear areas	37
PICN	1 200 000 loading cycles	PICN showed lower vertical loss than lithium disilicate and zirconia-reinforced variants	PICN materials possess suitable wear resistance for clinical use	38
PICN	At each evaluation (every year up to 5 years), replicates of the restorations were scanned to calculate material wear, with a concurrent clinical evaluation of the restorations	The 5-year restoration survival was 99.48%, and 90.62% successful, with minor defects. For the occlusal contact area, the estimated mean wear of the material was −27.97 μm	PICN restorations exhibit non-invasive characteristics toward human tissue	39
RNC (Lava ultimate)^b^	Chewing simulation, cyclic loading	Despite its suboptimal wear resistance, the material exhibits minimal detrimental effects on neighboring dentition	Chairside milling with new ceramic resins offers a superior alternative to traditional ceramics	40
RNC	A randomized clinical trial testing	Vertical wear rates per month: Tetric-C/EC 1.4 μm, Gradia-DP 1.8 μm. Volume wear rates per month: Tetric-EC 0.017–0.011 mm^3^, Gradia-DP 0.018 mm^3^	A 5-year study found comparable wear resistance among nano-, micro-filled, and conventional hybrids	41
RNC (ceram X)	Forty patients, each with four class I and II restorations under occlusion, were enrolled in this study	Minimal changes were observed in I&II occlusal restorations over 3 years: no performance differences from baseline for all materials	Further investigation is warranted to conclusively determine the long-term wear properties of RNC materials	42

a PICN: testing for durability with 1.2 million cycles and evaluating its impact on patient-reported outcomes over two years, with an emphasis on mimicking the physical properties of natural dental tissues.

b RNC: compared with PICN in a clinical trial of 40 patients, revealing unique responses to occlusal forces in class I and II restorations.

PICN (polymer-infiltrated ceramic network) materials represent an innovative blend of resin and zirconia enamel, consisting of a sintered ceramic matrix (86% by weight) infiltrated with a polymer matrix (14% by weight).^36^ Indications for PICN use include minimally invasive restorations, posterior crowns, veneers, inlays, on lays for posterior teeth, and implant-supported crowns.^36^*In vitro* studies^37^ indicate that although PICN exhibits greater wear depth than enamel, it is less prone to cracking within wear tracks. This suggests that PICN helps ensure the material remains intact and functional over time. Compared to lithium disilicate and zirconia-reinforced lithium disilicate, PICN showed less vertical loss after extensive loading cycles,^38^ suggesting promising wear resistance for clinical use. Although PICN is generally considered wear-resistant, the biocompatibility of particles released through wear requires further investigation. In a prospective clinical study^39^ spanning 5 years, the wear of PICN restorative materials was significantly lower, with a success rate of 90.62%. These findings indicate that PICN restorations exhibit noninvasive characteristics toward human tissues, providing a promising avenue for dental restoration without compromising tissue integrity. Overall, PICN materials exhibit commendable abrasion resistance; however, the health implications of particles produced after wear warrant further clinical investigation.

In contrast to PICN, resin nanoceramics (RNC) comprise a resin matrix integrated with nano-zirconia. Lava ultimate, a nanomaterial-reinforced dental composite resin, demonstrated suboptimal wear resistance *in vitro*, but it can cause minimal damage to opposing teeth^40^ In contrast, some researchers^41^ argue that there is no significant difference in wear resistance across nano-filled, micro-filled, and conventional hybrid composites based on a 5-year clinical trial. However, studies on chairside milling on ceramic resin^42^ caution against drawing definitive conclusions about wear properties from these studies, although they acknowledge that no RNC materials showed unacceptable wear patterns over a 3-year evaluation.^42^ In summary, while resin–zirconia RMC (resin-matrix ceramic) display satisfactory abrasion resistance, ongoing clinical research is essential for definitive validation.

PICN and zirconia CAD/CAM materials showed wear resistance that seems appropriate for clinical application in some research. Although the wear resistance of PICN may be lower than that of zirconia reinforced lithium disilicate and other zirconia materials, this is not necessarily a bad thing.^43,44^ In a study,^45^ compared with nanohybrid resin based composite (CO) and polymer filtered network ceramic (PINC), cubic zirconia (ZR) causes significantly higher enamel wear because ZR has better wear resistance. PINC tends to retain antagonist enamel, but at the cost of higher wear and tear on itself. In several other studies on composite materials, all resin-based materials showed minimal wear of antagonist enamel.^46,47^ Compared to dental enamel, these materials have poorer fracture toughness, resulting in fatigue failure before the enamel itself.^48^ In contrast, different authors found different results,^49,50^ showing how zirconia produced less antagonist wear in comparison with PINC materials. These inconsistencies may be related to the use of different testing procedures and different polishing treatments for each material, so more research data is needed in this area to provide clinical doctors with more novel and effective repair material choices.

### 2.4 Aging resistance

The antiaging properties of dental materials play a vital role in their durability and safety in the oral environment. This research focused on the aging resistance characteristics of resin–zirconia RMC (resin-matrix ceramic) ceramic materials. The effects of thermocycling on composite resin materials were shown in a clinical investigation,^51^ which indicated that thermocycling significantly affects the mechanical properties of these materials, particularly the hardness of martensite and edge chipping resistance (ECR). However, while aged PICN showed increased hardness and brittleness, its edge-chipping resistance remained stable. This phenomenon suggests that although PICN may become more rigid and brittle with age, it maintains its resistance to edge chipping.

In clinical practice,^31^ PICN is widely used for various applications, including single crown restorations, veneer restorations, inlays, and partial crown restorations. A 3-year cohort study on 76 Vita enamel single-crown restorations reported a survival rate of 93.9% and a success rate of 92.7%, based on the United States Department of Public Health Services (USPHS) evaluation criteria. During the follow-up process, secondary caries or debonding were not observed, and the main failure mode was restoration damage. The restoration showed good performance in color matching, anatomical shape, and edge fitting, which indirectly proves that the composite has good anti-aging performance.^31^

A 5-year clinical study^52^ on the performance of minimally invasive PICN restorations concluded that PICN restorations showed favorable clinical performance during this period. This suggests that PICN can maintain good clinical condition after five years of aging. However, further clinical evidence is required to assess the long-term aging resistance of this material. Resin–zirconia RMC (resin-matrix ceramic) has shown promising resistance to wear, tear, and aging but additional long-term clinical studies are necessary to fully validate their performance and reliability.

## 3 Biocompatibility of resin–zirconia RMC (resin-matrix ceramic) materials

### 3.1 Advantages of resin–zirconia RMC (resin-matrix ceramic) in inhibiting plaque formation

Plaque adhesion triggers the inflammatory response; therefore, reducing plaque adhesion on the surface of biomaterials can effectively reduce the inflammatory response. The formation of dental plaque biofilm occurs in three stages: acquired biofilm formation, bacterial adhesion, and plaque maturation. The surface adhesion of *Streptococcus mutans*, a key player in this process, includes two stages.^53^ In the early stage of adhesion, there is weak adhesion between bacterial cell wall proteins and acquired membrane sialo glycans. Subsequently, glucan adheres and binds to cell-surface receptors as ligands. Similar composite materials can inhibit the initial adhesion of bacteria,^54,55^ and resin–zirconia RMC (resin-matrix ceramic) materials exhibit excellent antibacterial performance after surface treatment. Using non-thermal atmospheric plasma treatment alters the surface elements and increases the surface energy of the material, resulting in a decrease in the adhesion of *Streptococcus mutans*.^56,57^ If fluoride is used for surface modification, it can also reduce the adhesion of *Streptococcus mutans*^58^ to suppress inflammation by inhibiting the formation of biofilms. In addition, experimental studies^59^ have shown that the zirconia component in resin–zirconia RMC (resin-matrix ceramic) positively affects oral bacterial adhesion. The response of human gingival fibroblasts (HGF) to linear KGGRGDSP and cyclic RGD_Fk_ sequences was compared, highlighting their influence on soft tissue sealing around implants. X-ray photoelectron spectroscopy and fluorescence microscopy were employed to verify polydopamine (PDA) deposition and covalent coupling of the Arg-Gly-Asp tripeptide complex (RGD). In addition, RGD complexes have a significant impact on the antibacterial properties of resin materials. On one hand, the surface of resin materials modified with RGD can alter the interaction between bacteria and the material surface. The adhesion proteins on the bacterial surface have difficulty binding to the modified surface, thereby reducing the initial adhesion of bacteria.^60^ Furthermore, when used in conjunction with antibacterial drugs or coatings, RGD modification can promote cell adhesion to the resin materials, allowing the material surface to be preferentially occupied by host cells, thus reducing the adhesion space for bacteria. At the same time, antibacterial drugs can act more effectively on the few adhered bacteria, enhancing the overall antibacterial effect. From the perspective of surface energy, RGD modification typically increases the surface energy of resin materials, improving the wettability of the materials. The enhancement of hydrophilicity is more conducive to cell adhesion, protein adsorption, and the uniform distribution of antibacterial drugs on the material surface, thereby enhancing the overall performance of the materials in biomedical environments.^61^ Thus, PDA–RGD-functionalized zirconia regulates specific HGF reactions while maintaining the antimicrobial activity of the PDA coatings. This surface-modified selective biological interaction model is expected to enhance soft-tissue integration around zirconia abutments by inhibiting plaque formation in clinical applications.

### 3.2 Positive effects of resin–zirconia RMC (resin-matrix ceramic) material on fibroblasts and related cells

Wound healing in oral soft tissue involves several types of cells, including fibroblasts (FBs), which play a vital role in coordinating with endothelial cells (ECs) to replenish the extracellular matrix (ECM).^62,63^ Due to the non-toxic effect of resin–zirconia RMC (resin-matrix ceramic) material on FBs, they maximize their potential, playing a crucial role in wound healing,^64^ particularly during cell proliferation and remodeling stages. During oral surgical wound healing, inflammatory cells release various mediators that stimulate and attract fibroblasts at the wound's border.^65^ Fibroblasts travel to the site of injury, where they produce collagen and fibronectin to facilitate the repair of ECM. FBs also play a role in granulation tissue.^65^

Several studies have shown that fibroblast proliferation can induce keratinocyte differentiation. The production of keratinocyte growth factor (KGF) by fibroblast was identified as key for these functions.^66^ The damage repair process can be accelerated by increasing the number of receptors on keratinocytes that correlate to specific signals, leading to enhanced differentiation of keratinocytes.^67^ Many regulatory molecules exert functions in the immune system and the skeleton, including cytokines, chemokines, receptors, and transcription factors.^68^ Thus, immune cells are crucial in maintaining bone homeostasis and regulating bone remodeling.^69^ By reducing the probability and progression of inflammatory reactions, effectively controlling osteolysis, and promoting bone formation. The resin–zirconia RMC (resin-matrix ceramic) did not affect the activity of many cells and could change the cell morphology (Table 2) when collagen was arranged in parallel. This facilitates tighter adherence of the mucosa to the implant base and crown, making it more difficult for microorganisms and plaques to adhere.^70^ As a result, resin–zirconia RMC (resin-matrix ceramic) material has become a preferred choice for implants.

**Table 2:** Resin–zirconia RMC (resin-matrix ceramic)'s cellular effects

Material	Cell line	Test	Effect	Ref.
Zirconia cercon base^a^	Human dental pulp stem cells (hDPSC)	Immunofluorescence and scanning electron microscope analyses	hDPSCs on zirconia: flat morphology, elongated processes	71
PICN^b^	Oral keratinocytes	Viability test	Cell viability peaked at 63.6% at 72 hours, accompanied by dense filopodial adherence to surfaces	72
PICN	Human gingival fibroblasts (HGFs)	Viability test and immunofluorescence staining	High viability (78.01%) and substantial cell proliferation (5356 ± 1580 cells at 72 h)	73
RNC (Lava ultimat)^c^	Human gingival fibroblasts (HGFs)	(1) Mitochondrial activity (XTT)	XTT, NRu, and CVDE remained largely unchanged from controls on days 1, 7, and 40, with NRu showing stability only on days 7 and 40	74
		(2) Membrane integrity (neutral red uptake, NRu)		
		(3) Cellular density (crystal violet dye exclusion, CVDE)		
PICN (Vita Enami)^d^	Human gingival epithelial cells		Smooth polishing of PICN material to *R*_a_ < 0.127 μm maximizes epithelial cell growth	75

a Zirconia cercon base: characterized in DPSCs *via* immunofluorescence and scanning electron microscopy.

b PICN: impact on oral keratinocytes and HGFs evaluated through viability testing and immunofluorescence staining.

c RNC (Lava ultimate): HGF metabolic activity was assessed *via* XTT, NRu, and CVDE assays.

d PICN (Vita Enamic): interaction with human gingival epithelial cells studied.

### 3.3 Mechanisms of resin–zirconia RMC (resin-matrix ceramic) material promoting bone integration

**Fig. 2:**
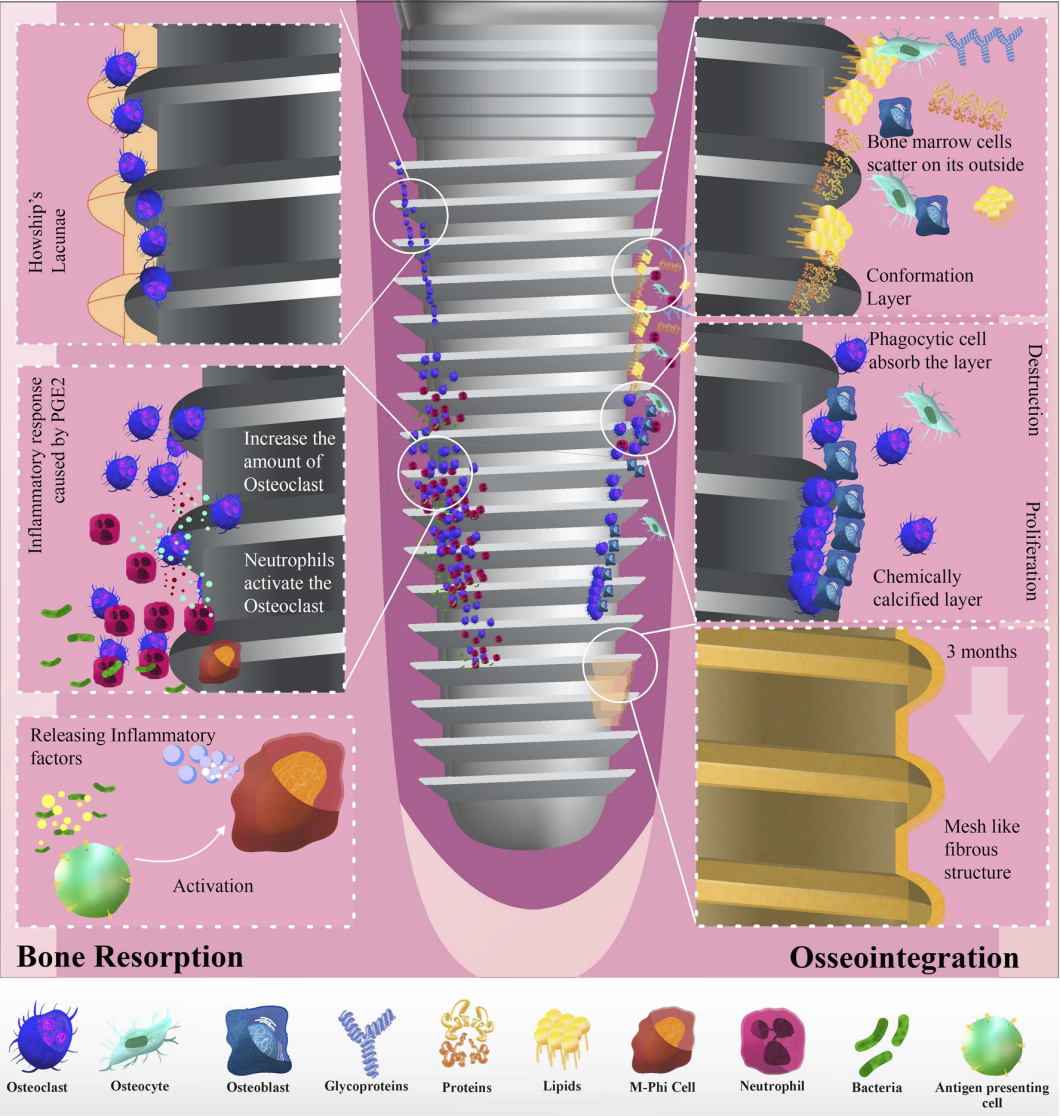
Schematic depicting alveolar bone resorption due to periodontitis at abutments.

Implantation of all materials was divided into three stages.^76^ Initially, the implant was covered with blood clots. Proteins, lipids, and glycoproteins from the bone marrow form a specific layer on the implant. Bone marrow cells covered the implant surface. In the second phase, phagocytic cells consume this conformational layer, precipitating hydroxyapatite and creating a chemically calcified layer. While some of these cells differentiate into osteoblasts, the remaining bone marrow cells organically attach to implants. Although trauma repair is the primary focus, bone synthesis and resorption occur simultaneously. Approximately, one month after the surgery, tissue death and growth begin. Implants often loosen due to bone tissue absorption from drilling, cutting, and pressure.^77^ The third stage begins when most phagocytic cells become osteoblasts and start biological mineralization. Collagen fibers form around the implant three months post-surgery, creating a mesh-like fibrous structure that leads to complete implant-bone integration (osseointegration) (Fig. 2).^78^

**Fig. 3:**
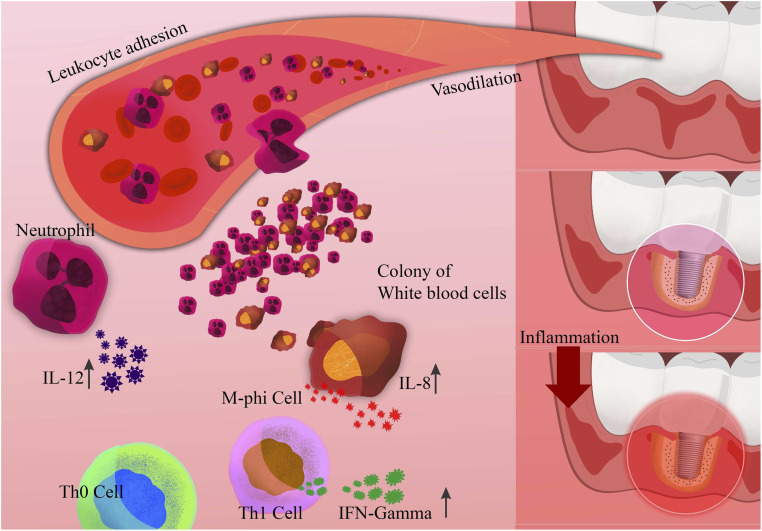
Schematic of periprosthetic inflammation and osteolysis induced by implantation.

Prostaglandins play a significant role in alveolar bone resorption, particularly in response to inflammatory stimuli. The inflammatory reaction sparked by prostaglandins leads to the infiltration of inflammatory cells, an increase in osteoclasts, and, ultimately, bone resorption.^79^ Neutrophils release cytokines and proteases such as IL-1, IL-6, and TNF-α, which, combined with macrophages, can activate osteoclasts and promote bone resorption. Certain bacterial products such as lipopolysaccharides (LPS) from the *Aggregatibacter* genus and non-endotoxic bone resorption agents can harm the alveolar bone (Fig. 3).^80^

Thus, zirconia-based (non-resin) surfaces have been shown to enhance osteoblast adhesion and endothelial responses; similar benefits are presumed when zirconia is incorporated within a resin-matrix ceramic, but direct evidence remains limited.^81,82^

Resin–zirconia RMC (resin-matrix ceramic) materials can also maintain implant stability through signaling factors, particularly the NO/cGmP and Wnt/β-catenin pathways.^83,84^ Preparation of porous zirconia material with nanotube structure by anodic oxidation, a material like the morphology of zirconia in resin–zirconia RMC (resin-matrix ceramic) materials. Researchers used western blot analysis to detect that the expression of p-ERK1/2 was promoted, and integrin β1 was detected. The expression of p-JNK, p-p38, CTSK, and TRAP was inhibited, and when osteoclasts were treated with p-FAK inhibitors, RT-qPCR showed significantly upregulated expression of OCN, OPN, ALP, and RuNX2. The expression of p-FAK was suppressed, and the expression of the osteoclast marker proteins CTSK and TRAP was correspondingly reduced. These results confirm that the material can inhibit osteoclast formation and modulate cytokine secretion through integrin-mediated FAK phosphorylation and the downstream mAPK pathway, making it more conducive to osteogenesis. Resin–zirconia RMC (resin-matrix ceramic) materials have great potential for application in bone-defect repair owing to their material properties.

### 3.4 The effect of resin–zirconia RMC (resin-matrix ceramic) material on the immune microenvironment

In clinical practice, the internal environment of the oral cavity is a key factor determining the applicability of oral materials. The damage of oral materials to the internal microbial community may lead to an imbalance in the oral microbial community, transforming the microbial community into related pathogen groups, which will increase the incidence of oral diseases, such as dental caries and periodontitis,^26^ ultimately affecting overall health. After implantation, chronic inflammation, tissue damage, and fibrosis may also occur. Therefore,^85^ it is essential to assess the impact of the resin–zirconia RMC (resin-matrix ceramic) material on the immune microenvironment and to explore the response of microorganisms in the oral environment. Through simulation and classification experiments, it was found that the resin–zirconia RMC (resin-matrix ceramic) material is insensitive to changes in the oral environment. It has high biological inertia and can maintain shape and structural integrity in acidic and alkaline oral environments.^86^ However, there is a lack of long-term *in vitro* and *in vivo* experiments on the immune microenvironment of the resin–zirconia RMC (resin-matrix ceramic) material, so further more explorations are needed.

## 4 Limitation of resin–zirconia RMC (resin-matrix ceramic) materials

Resin–zirconia RMC (resin-matrix ceramic) are susceptible to water absorption and degradation, which compromise their structural integrity.^87^ Water molecules infiltrate the resin matrix, leading to volumetric expansion. Hydrogen bonding between polymer carbonyl groups and absorbed water molecules facilitates plasticization, reducing the material's mechanical resilience.^88^ A decline in glass transition temperature (TG) induces the polymer's transition from a glassy to a rubbery state, enhancing flexibility and deformation capacity while compromising mechanical strength, rendering the material more susceptible to structural instability. Prolonged water exposure further reduces the material's thermal stability over time.^89^ These detrimental effects undermine the suitability of the material for dental restorations, which require high hardness and stability to endure occlusal forces. Furthermore, esterases in saliva or secreted by bacteria catalyze the hydrolysis of ester bonds in dental resin composites, progressively compromising the integrity of the resin–dentin interface and diminishing its mechanical properties.^90^ Water infiltration weakens the filler–resin interfacial adhesion, expediting filler particle debonding. Moisture-induced crack growth sensitivity exacerbates microcrack propagation along the filler interface or within the resin matrix, accelerating fatigue failure and increasing the risk of long-term fracture in restorations.^90^ While different fillers exhibit varying degrees of susceptibility to this degradation, the issue is intrinsic to resin-based materials and remains largely unavoidable.^91,92^ Consequently, if the service life of resin–zirconia RMC (resin-matrix ceramic) materials is extended in humid oral environments, the failure caused by degradation can be minimized to the greatest extent possible.

In the oral environment, we not only need to consider humidity, but also the ability of the mouth to withstand different temperatures. The normal temperature of the oral cavity is between 35 °C and 37 °C. However, this will change when consuming or drinking hot or cold substances. These temperature changes can cause expansion and contraction of the repair material, leading to the formation and propagation of mechanical stress and cracks.^92,93^ Due to the different thermal expansion properties of resin matrix and filler particles, repair materials containing resin are particularly susceptible to this type of fatigue.^94,95^ Research has shown that after 10 000 cycles of thermal cycling treatment on all study specimens, the fracture properties of polymer infiltrated ceramic networks (PICN) and resin nanoceramics (RNC) are significantly higher than those of lithium disilicate (LS) and zirconia reinforced lithium silicate (ZLS).^96^ So in future research, continuously exploring the optimal ratio of inorganic fillers to resin matrix to combat material failure is the direction of research.

## 5 Resin–zirconia RMC (resin-matrix ceramic) material in clinical applications

From a chairside standpoint, the resilience of resin-matrix ceramics translates to simplified intra-oral adjustments, faster polishing and notably lower antagonist enamel wear compared with lithium-disilicate glass-ceramics. These practical advantages shorten appointment times and reduce postoperative sensitivity—outcomes highly valued by clinicians and patients alike.^76,97^

Resin–zirconia RMC (resin-matrix ceramic) is widely used in clinical practice. Currently, the application of dental materials in the later stages of root canal treatment mainly relies on resin materials. The advantage of resin composite ceramic technology is that it has low requirements for cavity shape and does not require preventive expansion procedures. However, these materials are prone to issues such as poor fixation, detachment, and micro-leakage of the filling material. To address these concerns, resin–zirconia RMC (resin-matrix ceramic) materials offer significant benefits. They are applied in the dental field due to their superior biocompatibility and stability compared to other materials. Furthermore, it has the advantages of semi-transparent colors, easy color matching, and good aesthetics.^98^

Applying CAD/CAM technology, which is extensively used in clinical procedures like full crowns, inlays, fixed bridges, and veneers, to the production of resin–zirconia RMC (resin-matrix ceramic) materials enhances the quality and efficiency of manufacturing this novel ceramic material.^99^ Resin–zirconia RMC (resin-matrix ceramic) material differs from both traditional all-ceramic material and the silicon oxide material used in standard all-ceramic material. By using zirconia, which has superior biocompatibility, these materials not only offer a more patient-friendly option for oral applications but also provide a solution to the issue of acidic corrosion commonly seen in traditional materials. In addition, the newly introduced CAD/CAM method in clinical practice can also better match resin zirconia material. For the application of various data information, it mainly refers to normal tooth data, effectively measures appearance data indicators, and comprehensively measures and analyzes the appearance data of adjacent teeth and crowns with the same name. This results in a more accurate design and application of high-inlay restorations, providing better outcomes for patients.^100^ A clinical study used PICN Vita Enamic and feldspar ceramic Vitalocs mark II to treat 101 cases of posterior tooth defects. High inlay repair was performed, and 3 years after implantation, the retention rates of Vita Enamic and Vitalocs mark II were as high as 97% and 90.7%, respectively, with no significant difference between the two.^101^

Recent laboratory data clarify why the measured mechanical profile of resin–zirconia RMCs translates into the chairside benefits noted above. Their dentine-like elastic modulus (≈10 GPa) and enamel-like surface hardness (≈3 GPa) place occlusal stress below the crack-initiation threshold while limiting wear on the opposing tooth, outcomes mirrored in the *in vivo* study that documented 60% less antagonist abrasion than lithium-disilicate restorations.^76^ At the same time, nano-zirconia toughening raises fracture toughness to about 2.5 MPa m^½^, allowing thinner margin designs without compromising fatigue resistance. This modulus-toughness balance explains the high three-year retention rate (97%) reported for PICN Vita Enamic in posterior high-inlays.^98^

Equally important, the hybrid surface chemistry moderates the immune micro-environment once the restoration is placed. *In vitro* macrophage assays show a 30–40% reduction in TNF-α and a concomitant rise in IL-10 when cells are cultured on resin–zirconia RMC discs compared with monolithic zirconia, reflecting an anti-inflammatory, M2-skewed phenotype.^99^ Clinically, this translates into faster soft-tissue maturation and reduced marginal erythema, which dovetails with the lower micro-leakage rates already attributed to RMC's low polymerisation shrinkage. CAD/CAM processing further enhances this biological performance by producing an intaglio roughness below 1 μm Ra, a level that diminishes initial bacterial adhesion and improves adhesive bond durability.^100^

In summary, resin–zirconia RMC (resin-matrix ceramic) materials have demonstrated high practicality in clinical applications. It has high repair performance, retention rate, clinical success rate, and high patient satisfaction. However, its long-term clinical applications require further investigation.

## 6 Conclusion

This review summarizes the current research on resin–zirconia RMC (resin-matrix ceramic), highlighting their mechanical properties, aesthetics, and biocompatibility, making them well-suited for teeth and periodontal tissues. In terms of mechanical properties, resin–zirconia RMC (resin-matrix ceramic) reduces wear and aging, minimize material damage, and alter stress distribution, contributing to an extended service life. Regarding aesthetics, the color difference between resin–zirconia RMC (resin-matrix ceramic) and natural teeth is within an acceptable range, ensuring functionality is not compromised due to incompatibility. In terms of biocompatibility, these composites help reduce plaque adhesion, inhibit inflammatory reactions and osteolysis, and promote bone regeneration, all which support tissue regeneration and repair. Resin–zirconia RMC (resin-matrix ceramic) has unique properties that aid in healing damaged tissues by regulating the behavior of endothelial cells and modulating interactions among immune cells, osteogenic-related cells, nerve cells, and endothelial cells. They also exert a significant regulatory effect on osteoclasts and fibroblasts.

Current research on resin–zirconia hybrid (resin-matrix ceramic) systems is still fragmented. Long-term biocompatibility data are scarce because almost all human clinical studies end at the 24-month mark, and the few animal investigations that exist favour rodent calvarial models rather than true load-bearing sites. Interface durability has been explored only under uni-axial bending fatigue; oblique and shear loading—common in posterior occlusion—remain virtually untested, and temperature- or pH-cycling protocols vary so widely that results cannot be compared. The low-temperature degradation (t → m phase transformation) of zirconia grains that are embedded inside the resin scaffold has not been monitored in a realistic oral ment, so clinicians still do not know whether routine sand-blasting or acidic cleansers accelerate grain breakdown. In the biological arena, nearly every biofilm study relies on a single bacterial species such as *Streptococcus mutans*, while macrophage-mediated oxidation and the resulting cytokine response have never been quantified. Finally, no clinical standardisation framework exists: there is no universal shade/thickness chart, laboratories devise their own curing and polishing schedules, and the relevant ISO standards (4049 and 6872) do not cover ceramic–polymer laminates.

Future work therefore needs to move beyond descriptive laboratory studies. A five-year, multi-centre cohort trial that uses a single, clearly defined preparation and bonding protocol and incorporates yearly OCT scans plus salivary inflammatory markers would yield clinically meaningful longevity data. Real-time electro-chemical tracking of the tetragonal-to-monoclinic conversion inside hybrid blocks, performed under cyclic pH and mechanical loading, is required to understand zirconia low-temperature degradation when grains are shielded by resin. A multi-species biofilm model that is co-cultured with macrophages should be developed to measure reactive-oxygen production, cytokine release and resin-matrix oxidation products on the same surface. Mechanical testing needs a unified fatigue regimen—for example an oblique, step-stress protocol combined with standardised thermocycling—to permit head-to-head comparisons among commercial brands. On the regulatory side, a new annex to ISO 6872 should specify allowable resin content, curing depth, porosity thresholds and translucency ranges for hybrid ceramics, accompanied by a colourimetric chart that links thermal firing and polishing recommendations to final shade. Materials science efforts ought to focus on smart interphase additives, such as thiourethane or dopamine-functionalised silanes, that can self-heal micro-cracks and chelate zirconium to slow hydrolytic attack; these additives should be validated under accelerated chemical, thermal and mechanical ageing. Finally, digital-workflow studies must determine the optimal scanner settings, milling paths and burr geometries that minimise subsurface damage before the sinter-infiltration step. Addressing these gaps through coordinated, standardised research will convert resin–zirconia hybrids from a promising laboratory concept into a reliably documented clinical option.

To further improve the clinical applications of resin–zirconia RMC (resin-matrix ceramic) materials, stomatological hospitals should continue to promote their use at all levels. The mechanisms underlying their healing effects are complex and involve multiple cell types, regulators, and signaling networks. Future laboratory and clinical studies should compare the performance of new resin–zirconia RMC (resin-matrix ceramic) with commonly used materials, following clinically appropriate design and production standards. Future iterations of resin–zirconia RMC (resin-matrix ceramic) should aim to further improve biocompatibility, clinical applications, mechanical properties, and aesthetics that meet patient expectations.

## Conflicts of interest

The authors declare no conflict of interest.

## Data Availability

No primary research results, software or code have been included and no new data were generated or analyzed as part of this review.
